# Nutrient removal from human fecal sludge digestate in full-scale biological filters

**DOI:** 10.1016/j.chemosphere.2020.127219

**Published:** 2020-10

**Authors:** Aaron A. Forbis-Stokes, Graham H. Miller, Armel Segretain, Felahasina Rabarison, Tojoniaina Andriambololona, Marc A. Deshusses

**Affiliations:** a Department of Civil & Environmental Engineering, Duke University, Durham, NC, USA; b Triangle Environmental Health Initiative, Durham, NC, USA; c Loowatt Ltd, London, England, UK; d Duke Global Health Institute, Duke University, Durham, NC, USA

**Keywords:** Nitrification, Denitrification, Trickling filter, Anaerobic filter, Effluent polishing, Field study

## Abstract

There is a great need for simple methods for digestate management for potential household sanitation systems based on anaerobic digestion of minimally diluted fecal waste in countries that lack safe sanitation. Herein, a full-scale three-stage filter for nitrogen and phosphorus removal from anaerobic digester effluent was implemented in Madagascar. It included a trickling filter with crushed charcoal (for aerobic nitrification), a submerged anaerobic filter with bamboo chips (for denitrification), and a submerged filter with scrap iron (for phosphorus removal). All filter materials were sourced locally. Three parallel replicate systems were operated in two sequential 8-week phases for a total of 16 continuous weeks. Though the influent feed was not as expected, with much of nitrogen in the feed coming in as organic N and not as NH_3_–N, the filters still removed 38–49% of total incoming nitrogen. The filters achieved high rates of nitrogen transformation along with removing solids (73–82% turbidity removal), chemical oxygen demand (67–75% removal), and phosphorus (31–50% removal). Overall, the reaction rates from this full-scale study were in line with previous lab-scale investigations with scaled-down systems, supporting their application in real-world scenarios. Based on this study, simple effluent filters can support nutrient removal for small-scale and onsite fecal sludge treatment systems.

## Highlights

• High-strength anaerobic digester effluent was treated in three-stage filters.
• Full-scale filters were constructed in Madagascar using all local materials.
• Crushed charcoal trickling filters achieved nitrification rates up to 0.13 kg_N_ m^−3^ d^−1^.
• Bamboo woodchip filters achieved denitrification rates up to 0.03 kg_N_ m^−3^ d^−1^.
• Scrap iron filter removed 126 g_P_ m^−3^ d^−1^ with 23.2 g_P_ kg^−1^_Fe_ capacity.

## Graphical abstract

**Figure:**
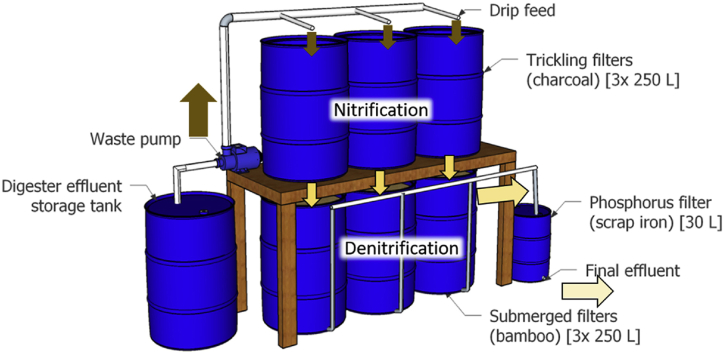

## 1 Introduction

Efforts towards onsite sanitation and fecal sludge treatment have intensified after shifting from the Millennium Development Goals, which focused on toilet access, to the Sustainable Development Goals (SDGs) which focus on safely managed sanitation. According to the SDGs, “safely managed” sanitation requires not only separating excreta from human contact at the source of defecation but also that fecal sludge from onsite facilities must be safely removed, treated, and disposed (United Nations, 2015). Septic tanks, cesspools and related onsite systems have widely been used for sanitation in rural areas, in both developed and developing countries. Anaerobic digestion is more recent and is becoming more common for household fecal waste treatment because of the energy benefits (Chaggu et al., 2007; Forbis-Stokes et al., 2016; Kujawa-Roeleveld et al., 2003; Lohri et al., 2010; Mang and Li, 2010; Tilmans et al., 2014). These technologies, however, only reduce solid and organic concentrations from the waste stream, resulting in effluents still high in nutrients and pathogens. Reuse of these effluents for agricultural purposes is feasible if adequately sterilized, but this is often not the case. Often, these effluents are disposed of in soil absorption or leach fields or in soak pits. In densely populated or flood prone areas and where reuse is not possible, these effluents need to be treated (i.e., organics and nutrients need to be removed and pathogens need to be sanitized) at the source in a relatively small footprint.

Compared to conventional wastewater treatment, effluents from onsite sanitation systems are more concentrated. This is particularly true for effluents from anaerobic digesters, which are often fed concentrated wastes to increase biogas yields and volumetric productivity. Thus reaching typical centralized wastewater treatment plants discharge standards (10–40 mg_BOD_ L^−1^, 10–50 mg_TSS_ L^−1^, 10–25 mg_TN_ L^−1^, 1–10 mg_P_ L^−1^) for effluents from anaerobic digestion of high-strength wastes (e.g., input concentrations ranging from 1000–5000 mg_BOD_ L^−1^, 5000–10,000 mg_TSS_ L^−1^, 1000–5000 mg_TN_ L^−1^, and 200–500 mg_P_ L^−1^) is costly and challenging. This would require 99% or greater reduction for some parameters which is not realistic (and may not be needed) for most onsite applications. This is why some of the new standards that are being developed, such as the recent ISO 30500 (International Organization for Standardization, 2018), have a focus on load reduction rather than effluent concentrations. Specifically, the ISO 30500 calls for 70% load reduction of total nitrogen and 80% load reduction for total phosphorous in non-sewered sanitation systems. Still, achieving removals of 70–80% can be challenging with limited resources.

Trickling filters are capable of providing combined organic removal and nitrification, but research on trickling filters in application of onsite wastewater treatment or anaerobic digester effluent is still fairly limited (Chernicharo and Nascimento, 2001; Forbis-Stokes et al., 2018; Khan et al., 2011; Tandukar et al., 2006; Vieira et al., 2013; von Sperling and Chernicharo, 2005; Xia et al., 2012). From these previous studies, some were either able to reach high removal percentages (70–90%) or treat high inlet concentrations, but with exception to Forbis-Stokes et al. (2018), none achieved both simultaneously. Research performed by (Forbis-Stokes et al., 2018) and Hunter and Deshusses (2019) evaluated different trickling filter media for their suitability to treat high strength wastewater and representative digestate (1900–4600 mg COD L^−1^; 500–3300 mg TN L^−1^; 1000–1400 mg PO_4_–P L^−1^), respectively and found that biochar, granular activated carbon (GAC), and zeolite can achieve high removal efficiencies for organics and turbidity, and high nitrification rates (0.075–0.252 kg_TAN_ m^−3^ d^−1^).

Woodchip as a medium for anaerobic filters for denitrification have garnered much interest recently (Addy et al., 2016; Bock et al., 2015, 2016; Christianson et al., 2017; Lynn et al., 2015a, 2015b; Pluer et al., 2016). Reactors in these previous studies were able to support denitrification for waste streams up to 30 mg NO_3_–N L^−1^ and at rates of 2–22 g N m^−3^ d^−1^. These reactors are beneficial in that an exogeneous electron donor (other than the woodchips) is not required for denitrification, providing low-cost and low-maintenance means for denitrification. Two studies have evaluated woodchip bioreactors for onsite sewage effluent (Rambags et al., 2016; Robertson et al., 2005) with success (87–99% from influent of ∼30 mg NO_3_–N L^−1^). Forbis-Stokes et al. (2018) performed a laboratory study with bamboo and eucalyptus woodchips to treat nitrified swine waste digestate at higher feed concentrations (299 mg NO_3_–N L^−1^) and achieved higher nitrate removal rates (66 g N m^−3^ d^−1^ for bamboo and 36 g N m^−3^ d^−1^ for eucalyptus). This study was later followed by a study with digestate from actual dog feces and human urine (Hunter and Deshusses, 2019) which found similar results (37 g N m^−3^ d^−1^ removal rate for bamboo filter treating 558 mg NO_3_–N L^−1^).

The latter two studies (Forbis-Stokes et al., 2018; Hunter and Deshusses, 2019) used two-stage biological filters to nitrify and denitrify high-strength waste from anaerobic digestion. In both cases, effective removal of COD, nitrogen and phosphorus was observed, and biochar and shredded bamboo were found to be effective packing for these filters. Findings from these studies were used to inform full-scale reactor design which is the subject of this manuscript. In this study, high-strength digester effluent was treated with the use of biological filters containing media and materials commonly found in developing countries. The first stage was a trickling filter to remove residual organics and nitrify ammonia, the second stage was a submerged anaerobic filter to denitrify nitrate produced in the first stage so that nitrogen was removed from the effluent, and the final stage was an iron filter to remove phosphates.

## 2 Materials and methods

### 2.1 Study design

This study took place at Loowatt LTD facilities in Antananarivo, Madagascar. Loowatt operates an anaerobic digester with a feed of fresh human waste (urine and feces) mixed with food waste (∼10% by volume, mainly banana peels) mixed in water. The digester is operated at ambient temperature (15–22 °C on average) with an approximate hydraulic residence time (HRT) of 90 days. The digester produces about 500 L of digestate per week collected in 330 Gallon (1250 L) Tote Tanks. For the purpose of this study, some of the anaerobic digester effluent was diverted daily to a 200 L barrel for use as substrate. A volume of about 70 L d^−1^ was treated in our filters. A peristaltic pump was used to feed the filters from the 200 L collection barrel. A filter tube consisting of a wrapped 1 mm mesh screen was installed at the inlet of the pump to strain large particles out of the digester effluent to prevent clogging in the pump lines. Digestate analysis before this experiment found the effluent contained, on average, 2510 mg TKN L^−1^; 2270 mg NH_4_–N L^−1^; 163 mg P L^−1^; and 2750 mg total organic carbon (TOC) L^−1^ (N = 15).

The test setup consisted of three identical treatment trains operated in parallel. Digestate from the feeding barrel was pumped to the top of the three trickling filters (TFs) operated in parallel using a peristaltic pump (Masterflex EW-07542-60, 50 rpm, fixed-speed drive). Three pump heads were installed (Masterflex EW-77200-60, L/S Easy-Load II) on a shared drive shaft so that three equal and parallel flow lines were used (Masterflex L/S 18, Food-Grade Tygon). To mimic dynamic conditions experienced in onsite sanitation, digestate was pumped ten times per day; the pumping rate was 0.19 L min^−1^ per filter. For the first 8 weeks (Period I), the pump was on for 125 min, for a total daily flow of 23.8 L filter^−1^ d^−1^. For the second 8 weeks (Period II), the pumps were on for 62.5 min for a total daily flow of 11.9 L filter^−1^ d^−1^.

Each of the pump flow lines was connected to one of three 250 L barrels used as trickling filters. Trickling filters were fed from the top where a plastic spreading cone was placed on the upper surface of the filter medium to more evenly distribute the flow. A sampling port was placed at the lowest point of the barrel (approximately 1.5 cm below the drains) so that a small volume could pool for sample collection. The effluent from each trickling filter was directed to a submerged anaerobic filter located directly below each of the trickling filters. The anaerobic filters were also 250 L barrels and also operated in a downflow mode. The outlet pipe at the bottom of each of the submerged anaerobic filters rose to the top of the barrels to maintain a water level 1–5 cm above the filter medium. The three submerged filter outlets were combined into one feed pipe for the phosphorus filter. All flow from the top of the trickling filters to the final effluent was induced by gravity.

### 2.2 Filter materials

Six 250 L barrels (HDPE drum, 101 cm height x 59 cm diameter) were used to make the trickling filters and submerged filters. The tops of the trickling filter barrels were fully removed for open airflow and covered with mosquito netting to protect from pests. The trickling filter barrels were packed with crushed charcoal found in local markets. The charcoal was crushed manually with a hammer and sieved before adding it into the drums. Particles between #4 and #8 mesh (2.38 mm and 4.76 mm) were used. The bottom 15 cm of the drum was filled with gravel to keep the filter medium from washing out and then packed with 66 cm of crushed charcoal. The effective treatment volume of each trickling filter was 180 L. Based on flow rates from Period I and II, the empty bed residence time (EBRT) was 7.6 days and 15.2 days, respectively.

The submerged anaerobic filters were filled with bamboo wood chips based on the success of the material in our previous studies (Forbis-Stokes et al., 2018; Hunter and Deshusses, 2019). Bamboo was locally sourced and shredded to chips approximately 25–100 mm long and 5–10 mm thick. These drums were also filled with 15 cm of gravel at the bottom to retain the filter medium and then filled to the top with bamboo chips. The effective treatment volume of each anaerobic filter was 232 L, resulting in EBRTs of 9.8 and 19.6 days for Period I and Period II, respectively. The phosphorus filter was a 50 L barrel and was filled with steel wool iron sponges and metal machining chips made of low-carbon steel, typically >98% iron by weight. Without the ability to characterize composition, mass was assumed to be fully iron. Previous researchers have used other sources of iron from acid mine drainage residual (68% Fe (Christianson et al., 2017)), steel slag (1.2% Fe (Christianson et al., 2017)), and ochre (65% Fe (Dobbie et al., 2009),) for similar purposes. Because effluent from all three parallel treatment trains entered the single phosphorus filter, the flow during Period I and Period II was 71.3 L d^−1^ and 35.6 L d^−1^, respectively, corresponding to EBRTs of 0.77 and 1.54 days, respectively.

Six days prior to packing in the filter drums, the trickling filter medium was washed with rainwater and pond water. Washing was performed to remove dust and small particules, and to wet and inoculate the charcoal. Local pond water was chosen for this due to the assumed presence of naturally occurring nitrifying and denitrifying bacteria. The water was not characterized due to resource limitations, but the nitrifying and denitrifying activity began soon after startup possibly confirming this assumption. Once the medium was packed in the barrels, the inoculation process was continued by providing a mix of 25% digester effluent and 75% pond water at a rate of 23.7 L filter^−1^ d^−1^ for three days prior to normal operation. An additional 12 L of aquarium water (as another nitrifying bacteria source) was added on the morning of the final inoculation day to increase the volume of the inoculation feed. During the inoculation period, the filters were also aerated periodically with a 3 L min^−1^ aquarium air pump in the same manner as described below, with a total air supply of 1038 L filter^−1^ day^−1^. Submerged filters were packed with bamboo chips and fully submerged in water on the same day that TFs were packed. The submerged filters were inoculated with trickling filter effluent while the trickling filters were being inoculated. The steel wool and metal machining chips used in the phosphorus filter were washed with water to remove any oil and grease.

The trickling filters were aerated using an aquarium pump (Bestgle 2.5 W 200 V Mini Aquarium Air Pump) with a 3 L min^−1^ capacity. Each trickling filter had its own air pump, in which the line was split into two inlets on the filter, plumbed to sit above the sample pool, as to prevent any liquid in the air lines. Each air pump was operated on the same interval of the effluent pump (10 times d^−1^), for a total of 346 min daily, for a daily air flow of 1038 L filter^−1^ day^−1^. Aeration pumps were triggered at the same time as the feed pump as to ensure aeration was on when the new feed entered the filter.

### 2.3 Experimental protocols and analytical methods

Once the inoculation was completed, feed into the system was switched to the digester effluent, and operating each filter at 23.7 L filter^−1^ d^−1^ for 8 weeks. At the end of the first 8-week treatment, performance was not as high as desired, and thus the feed was reduced by 50% for the last 8 weeks of operation. Between these two phases, the trickling filter media was gently washed with tap water to slough off debris without fully washing out the biofilm that had developed. The top 10 cm with the greatest amount of clogging was removed and washed while the remaining filtration volume was washed while in the drum. No filter media was replaced or regenerated so that longevity over the entire 16 week study could be evaluated.

Effluent samples were taken from the anaerobic digester, i.e., the trickling filter influent, trickling filter, submerged filter, and phosphorus filter effluents weekly. These samples were analyzed for total nitrogen (TN), total ammonia nitrogen (TAN or NH_3_–N), nitrate (NO_3_–N), nitrite (NO_2_–N), total phosphorus (P), chemical oxygen demand (COD), five-day biochemical oxygen demand (BOD_5_), total suspended solids (TSS), turbidity (NTU), and pH. Not all sampling locations were analyzed for all parameters each week because of limited resources and analytical capabilities. Instead, one of the three trickling and submerged filters was sampled each week in a rotating fashion such that each was sampled every 3 weeks. Every 4 weeks, all trickling filters were sampled simultaneously and analyzed for key parameters, turbidity, NH_3_–N, and NO_3_–N, while all submerged filters were sampled and analyzed for turbidity and NO_3_–N. Additionally, BOD_5_ and TSS were only analyzed every 4 weeks as it was assumed that these parameters had a proportional removal relationship to COD and turbidity, respectively. All analyses were performed at national labs located in Antananarivo.

## 3 Results

**Fig. 1:**
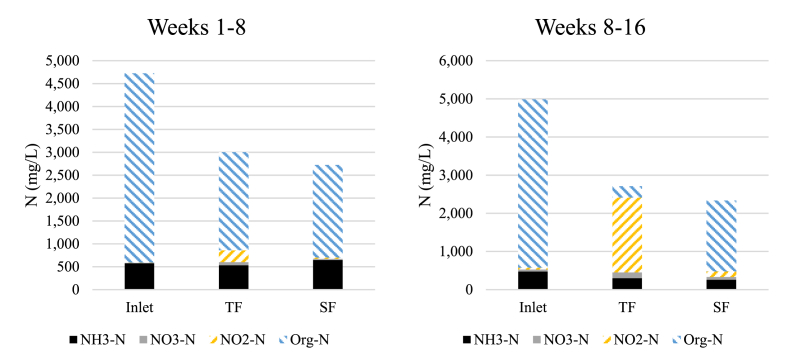
Nitrogen speciation for filter inlet and outlets of the trickling filter (TF) and submerged filter (SF) from Phase I (weeks 1–8, left) and Phase II (weeks 8–16, right) of this study.

Average values for nitrogen speciation in Weeks 1–8 and 8–16 are shown in Fig. 1, and average values for all parameters are shown in Table 1, Table 2. In general, the three replicate systems performed similarly with moderate variations (±10%, results not shown) in their performance. Those variations were attributed to small differences in conditions, flow distribution and alike, inherent to field research. The total nitrogen concentration fed to the filters was greater than expected while the percent of total nitrogen as ammonia nitrogen was much lower than expected (previous effluent results from the same digester were 2510 mg TKN L^−1^ and 2270 mg NH_4_–N L^−1^ while our study was 4750 mg TKN L^−1^ and 480 mg NH_3_–N L^−1^). TKN was not measured in our study; however, organic-N was calculated as the sum of NH_3_–N, NO_3_–N, and NO_2_–N subtracted from total N. The much greater concentration of organic N than expected had major impacts on the desired performance of the filters. The trickling filters had been designed for expected digestate characteristics with the vast majority of incoming N being in the form of NH_4_^+^/NH_3_ which would then be directly available for conversion into NO_2_^-^ or NO_3_^-^ by nitrification, before being denitrified to N_2_ in the submerged filters. Instead, with N primarily as organic nitrogen (org-N), it must first undergo ammonification (where NH_2_ groups are converted by bacteria into NH_3_) in the trickling filters, producing NH_3_ prior to nitrification, thus adding a possible rate limiting step to the process. Organic N physical removal by the filter medium is possible. However, since the organic N remained in contact with the biofilm, it was assumed that all organic N would eventually be transformed into NH_3_ via ammonification. The EBRT of the trickling filters (7.6 d) was designed according to the minimum residence time for ammonium oxidizers (1.5–2.1 d) and nitrite oxidizers (1.4–1.9 d) (Rittmann and McCarty, 2001). Ammonification rates are not as well studied as nitrification, but Stefanakis et al. (2014) reported a rate of 0.53 g N m^−2^ d^−1^ where the m^−2^ is the applied surface area in a vertical flow constructed wetland. This rate is similar to ammonification in trickling filters, 0.5–0.8 g N m^−2^ d^−1^ (Rittmann and McCarty, 2001). The additional reaction time required for ammonification, as well as the oxygen consumed probably limited the ability of the trickling filters to fully nitrify the incoming organic N.

**Table 1:** Summary of average, standard deviation, percent change for each stage of filtration and number of observations (N) for each stage of filtration during weeks 1–8. Percent change for each stage is in reference to the input to that stage. Units are mg L^−1^ unless otherwise specified. Cells without values are because no measurement was made for the given species post phosphorus filter.

	Inlet			Trickling filters				Submerged filters				Phosphorus filter			
	Avg.	St. Dev.	N	Avg.	St. Dev.	Δ%	N	Avg.	St. Dev.	Δ%	N	Avg.	St. Dev.	Δ%	N
Total N	4713	1093	8	3004	970	−36%	8	2564	1108	−15%	8	2902	1178	13%	8
NH_3_–N	575	495	8	531	510	−8%	8	645	514	21%	8				
NO_3_–N	10	7	3	66	42	5	8	29	5	−57%	8				
NO_2_–N	3	5	3	261	245	82	8	27	14	−90%	8				
Total P	224	51	8	139	59	−38%	3	208	139	49%	8	111	59	−47%	8
pH (−)	8.1	0.2	6	8.1	0.9	0%	6	7.8	0.3	−4%	6	7.9	0.3	1%	5
COD	10,984	3859	8	5704	1100	−48%	8	4143	735	−27%	8	2727	810	−34%	3
BOD_5_	1296	204	2	910	433	−30%	2	2260	2351	148%	2				
TSS	8217	1487	3	4400	700	−46%	3	3883	797	−12%	3	3533	1712	−9%	3
Turbidity (FTU)	3383	1815	8	1340	392	−60%	3	767	158	−43%	3	903	431	18%	3

**Table 2:** Summary of average, standard deviation, percent change for each stage of filtration, and number of observations (N) for each stage of filtration during weeks 9–16. Percent change for each stage is in reference to the input to that stage. Units are mg L^−1^ unless otherwise specified. Cells without values are because no measurement was made for the given species post phosphorus filter.

	Inlet			Trickling filters				Submerged filters				Phosphorus filter			
	Avg.	St. Dev.	N	Avg.	St. Dev.	Δ%	N	Avg.	St. Dev.	Δ%	N	Avg.	St. Dev.	Δ%	N
Total N	4800	198	8	2690	661	−44%	8	2463	345	−8%	8	2467	223	0%	8
NH_3_–N	474	66	8	299	84	−37%	8	255	77	−15%	8				
NO_3_–N	56	10	2	155	83	177%	8	76	78	−51%	8				
NO_2_–N	47	20	2	1957	3612	4093%	8	146	226	−93%	8				
Total P	231	17	8	202	33	−13%	2	173	16	−14%	8	158	12	−9%	8
pH (−)	8.3	0.1	2	8.1	0.3	−2%	2	8.4	0.0	4%	2	8.5	0.0	1%	2
COD	8051	979	8	6039	547	−25%	8	3077	389	−49%	8	2625	177	−15%	2
BOD_5_	900	1	2	600	286	−33%	2	422	333	−30%	2				
TSS	5550	354	2	4203	499	−24%	2	2138	725	−49%	2	1958	1191	−8%	2
Turbidity (FTU)	2524	495	8	1375	291	−45%	3	660	268	−52%	3	442	128	−33%	3

Because NH_3_ is produced by ammonification and consumed by nitrification, interpreting nitrification and denitrification rates is difficult based purely on inlet and outlet concentrations of NH_3_, NO_2_^−^ and NO_3_^−^. Rates, therefore, were calculated based on a mass balance of N as shown in Table 3. The mass balances for nitrogen in the system were based on the following assumptions: 1) The increase in NO_2_–N + NO_3_–N was equal to the decrease in NH_3_–N through nitrification; 2) The decrease in org-N from inlet to outlet produced NH_3_–N through ammonification; 3) The decrease in TN from inlet to outlet of the trickling filters was only through NH_3_ stripping or NH_4_ adsorption. These assumptions were checked by comparing the measured trickling filters effluent NH_3_–N concentration to the theoretical NH_3_–N recovered based on the inlet NH_3_–N (from measured NH_3_–N and formed NH_3_–N through ammonification) minus the NH_3_–N removed (from NO_2_/NO_3_ formation or TN lost). Average concentrations in Phase I and Phase II were multiplied by the corresponding flow rates to perform calculations in units of g d^−1^ and are reported in Table 3.

**Table 3:** Mass balance of NH_3_–N calculations and results for Phase I and Phase II of the study.

Process	Calculation	Phase I [g d^−1^]	Phase II [g d^−1^]
NH_3_–N loading	Measured value	13.7	5.6
NH_3_–N produced via ammonification	Organic N in – out	47.3	48.8
Total NH_3_–N loading	NH_3_-N_in,measured_ + NH_3_-N_produced_	61.0	54.4
NH_3_–N transformed via nitrification	Increase in NO_2_–N and NO_3_–N	7.5	23.9
Total N in	Measured value	111.9	57.0
Total N out	Measured value	71.3	31.9
NH_3_–N removed from system via adsorption or stripping	Total N in – out	40.6	25.1
NH_3_–N calculated in outlet	Total NH_3_–N loading – transformed – removed	13.0	5.5
NH_3_–N in outlet	Measured value	12.6	3.6
Mass balance accuracy	(Calculated – Measured)/Calculated	97%	65%
NH_3_–N reduction	1 – (Total in – Out)/Total in	79%	90%

Closing the mass balance for data from Weeks 1–8 was very accurate (97%) while for Weeks 9–16, it was less accurate (65%) (Table 3). The difference may be due to the high standard deviation of data in Period II, particularly for NO_2_–N (Table 2). Even so, the calculated total NH_3_–N reduction rate was nearly the same for both periods, 0.241 and 0.245 kg_N_ m^−3^ d^−1^. There was a shift in the dominating reduction mechanism between the two phases, however. In Phase I the N removal rate via adsorption or stripping was 0.225 kg_N_ m^−3^ d^−1^ compared to 0.139 kg_N_ m^−3^ d^−1^ in Phase II. The decreased removal rate, though not statistically significant due to variance (*P* = 0.22), may have been due to lessened adsorption capacity of the crushed charcoal over time. The conversion by nitrification (remaining NH_3_–N after subtracting removed NH_3_–N divided by NO_2_–N and NO_3_–N produced) increased from 37% to 81% between Phase I and II (0.04 and 0.13 kg_N_ m^−3^ d^−1^). The decreased loading rate in Phase II allowed increased reaction times for nitrifying bacteria. The percent reduction of NH_3_–N was also greater in the second phase, 90% versus 79%.

Nitrite and nitrate removal performance in the submerged filters remained high through both Periods I and II. In Period I, 37.4 mg NO_3_–N L^−1^ and 234 mg NO_2_–N L^−1^ were removed, and 78.3 mg NO_3_–N L^−1^ and 1811 mg NO_2_–N L^−1^ were removed in Period II. The rates of removal in Period I were 0.004 kg NO_3_–N m^−3^ d^−1^ and 0.024 kg NO_2_–N m^−3^ d^−1^ (total of 0.028 kg_N_ m^−3^ d^−1^). The rates of removal in Period II were 0.004 kg NO_3_–N m^−3^ d^−1^ and 0.093 kg NO_2_–N m^−3^ d^−1^ (total of 0.097 kg_N_ m^−3^ d^−1^) Total denitrification rates (removal of both NO_3_–N and NO_2_–N) were then 0.028 kg_N_ m^−3^ d^−1^ and 0.097 kg_N_ m^−3^ d^−1^. A generalized ratio of mass of BOD required per mass of NO_3_–N is 4:1 (Rittmann and McCarty, 2001). The ratio decreases by 40% when starting from NO_2_–N based on a stoichiometry derived from electron balances. Based on these ratios, initial concentrations of NO_3_–N and NO_2_–N in Period I and Period II required 21.2 and 63.2 g_BOD_ d^−1^, respectively. COD was analyzed more frequently than BOD due to lab constraints (total N = 16 vs. 4). The resulting relative standard deviation of COD in reference to average value was much lower than BOD, indicating a more accurate value. During these periods, 1561 and 2962 mg_COD_ L^−1^ were removed from the trickling filter effluent, corresponding to 37.1 and 35.2 g_COD_ d^−1^. If COD were used as a substitute for BOD in the required carbon ratio for denitrification, it is possible that all denitrification was enabled by residual organics in the TF effluent for Phase I. In Period II, however, more COD was required to enable the measured denitrification than was in the TF effluent (63.2 g_COD_ d^−1^ required, 35.2 g_COD_ d^−1^ removed from TF effluent). The woodchips then provided at least 28.0 g_COD_ d^−1^ in this period. Using a 4:1 ratio for COD:NO_3_–N, the woodchips alone supported a removal rate of 0.030 kg_N_ m^−3^ d^−1^ in Period II.

**Fig. 2:**
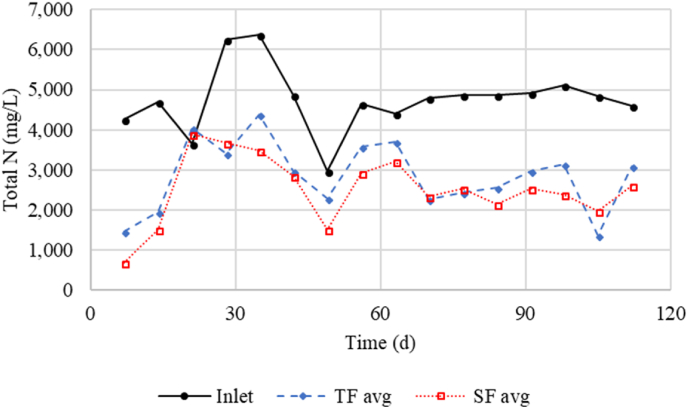
Total N concentrations from filters inlet, trickling filters (TF) outlet, and submerged filters (SF) outlet. TF and SF values are averages from outlets of the three filters at those stages.

When considering BOD, though a less reliable value in this study, the increased concentration between inlet and outlet of the submerged filter in Phase I (Table 1) indicates that excess BOD was produced than was required for denitrification. The production of BOD was most likely due to more BOD leached from woodchips than consumed in denitrification. This increase supports the finding that the trickling filter effluent had sufficient residual organics to support denitrification during Phase I. The BOD removal rate in Phase II was 2.11 g d^−1^ (Table 2), much less than COD. Based on the BOD removal value, wood chips would have been required to provide at least 61.1 g_BOD_ d^−1^ to enable denitrification. With a loading of 1.84 g NO_3_–N and 23.25 g NO_2_–N per day, 7.36 and 55.8 g_BOD_ was required per day for complete denitrification (total of 63.2 g d^−1^). The 61.1 g provided by wood chips then accounts for 96.7% of BOD demand in the trickling filter effluent. By providing 61.1 g_BOD_ d^−1^ and using the 4:1 BOD:NO_3_–N ratio, the wood chips alone could have provided a removal rate of 0.066 kg_N_ m^−3^ d^−1^ of denitrification in Phase II. Submerged filter effluent concentrations of NO_3_ and NO_2_ did not show any increasing trends towards the end of the study, indicating that the woodchips could still provide sufficient reducing equivalents for denitrification (see Fig. 2).

**Fig. 3:**
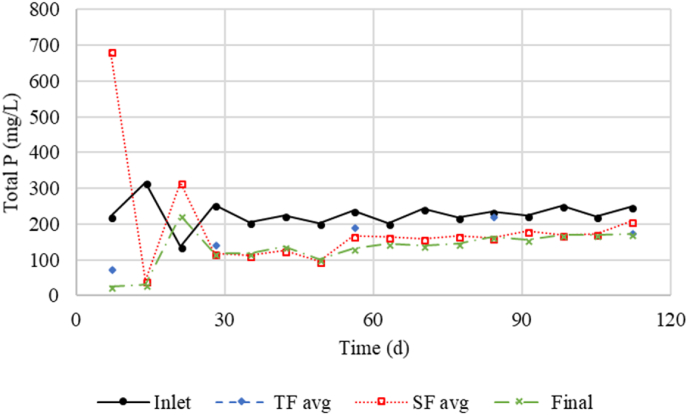
Total P concentrations from filters inlet, trickling filters (TF) and submerged filters (SF) outlets. TF and SF values are averages from outlets of the three filters at those stages.

Phosphorus removal in the final filter was much higher in Period I (47%) than in Period II (9%), which was reflected by an increase in average effluent concentrations, 111 and 158 mg P L^−1^ (*P* = 0.04), respectively (Table 2, Table 3). The incoming concentration as well as flow rate from the submerged filters were on average lower in Period II. With these factors in mind, P removal was expected to be greater. The decreased P removal may be evidence of decreased removal capacity of the iron scrubber. Further evidence of the decreased capacity can be seen in Fig. 3 where the final effluent concentration visually increases at a slow rate over the duration of the study. Detailed examination of Fig. 3 reveals that the trends in P removal in the trickling or the submerged filters were not straightforward. During Period I, the phosphorus filter caused an increase in the liquid’s turbidity. It was also noticed that the phosphorus filter added a dark red color to the liquid. Between Period I and II the phosphorus filter was washed with tap water. After this, the turbidity of the effluent decreased, but some red color was still leached (Fig. 3). A total of 129 g of P was removed in Period I and 10 g in Period II. The mass of material used in the phosphorus filter was 6 kg. Assuming that the material was 100% iron, the removal capacity was 23.2 g_P_ kg_iron_^−1^. The removal rates for Period I and Period II were 0.126 and 0.010 kg P m^−3^ d^−1^, respectively.

**Fig. 4:**
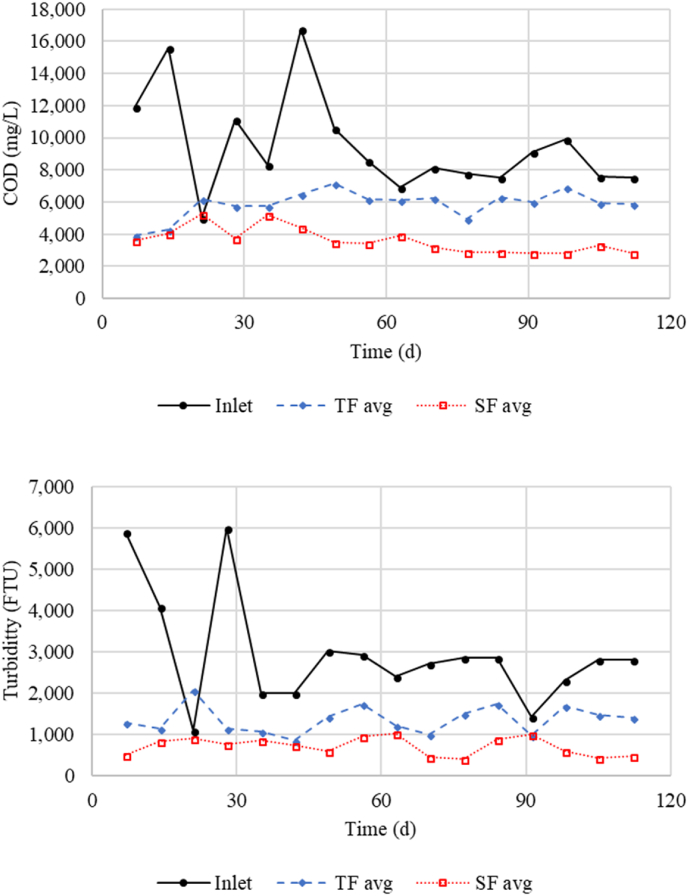
Time-series plots of filter inlet, trickling filter (TF) outlet, and submerged filter (SF) outlet COD (top) and turbidity (bottom) for the full study duration.

The cumulative treatment performance for Period I resulted in 38% removal of TN, 50% removal of P, 75% removal of COD, and 73% removal of turbidity (Table 1). For Period II the performance changed to 49% removal of TN, 31% removal of P, 67% removal of COD, and 82% removal of turbidity (Table 2). Inlet concentrations were similar during both periods except for higher concentrations of COD (11,000 mg L^−1^ Period I and 8100 mg L^−1^ Period II) and turbidity (3400 NTU Period I and 2500 NTU Period II) in Period I. The decreased COD removal was likely due to decreased loading, and the final COD concentrations were similar for both periods. The decreased P removal was likely due to consumption of iron as previously discussed. The final effluent did not show any increasing trends in COD or turbidity during the study (Fig. 4), showing that solids and organics removal did not decline over time. In fact, the COD concentration had a decreasing trend from day 35 onward. While trickling filter effluent COD and turbidity typically fluctuated in response inlet variations, the submerged filter effluent remained fairly consistent (Fig. 4).

## 4 Discussion

The much greater than expected concentration of organic N in the effluent of the anaerobic digester had major impacts on the performance of the filters. The nitrogen loading to the anaerobic digester that served as feeder for this study was high, particularly due to large volume of human urine, but the nitrogen from this source was expected to be converted to NH_3_ given the long HRT (90 days) in that anaerobic digester. Approximately 85% of total N was organic N, whereas it is more typical to see 75–90% of total N in the form of NH_3_–N in digester effluents. The reason for the high organic N concentration is not certain, but it may be due to biomass carry over from the anaerobic digester after being in operation with high-strength loading for a long period of time. Because of the high organic N concentration, the NH_3_–N reduction rate had to be calculated based on mass balances. The calculated rates from both periods (0.241 and 0.245 kg_N_ m^−3^ d^−1^) were quite high compared to previous studies (Forbis-Stokes et al., 2018, achieved 0.102 kg_N_ m^−3^ d^−1^). The referenced NH_3_–N removal rate was primarily due to nitrification, though. When considering only nitrification and not adsorption or stripping, Phase I and Phase II achieved rates of 0.041 and 0.133 kg_N_ m^−3^ d^−1^, respectively. The low nitrification rate in Phase I may have been due to overloading the filters. Phase II had a threefold greater nitrification rate under half of the loading rate in reference to Phase I and still 1.6 to 10 times greater than the referenced literature (usual rates are 0.010–0.081 kg_N_ m^−3^ d^−1^) (Aiyuk et al., 2004; Berger, 2012; Vieira et al., 2013). The results show that high NH_3_ removal rates can be obtained under very concentrated loading conditions with simple materials.

Total denitrification rates (removal of both NO_3_–N and NO_2_–N) were 0.028 kg_N_ m^−3^ d^−1^ and 0.097 kg_N_ m^−3^ d^−1^. The rates in Phase II were higher than what was shown in previously referenced work on woodchip bioreactors with exception to Forbis-Stokes et al. (2018) rate of 0.066 kg_N_ m^−3^ d^−1^. The Period I removal rate was likely lower due to the low influent NO_2_–N and NO_3_–N concentrations, while the Period II rate was likely higher due to higher influent COD. This study had a higher organic concentration entering the submerged filters than the filters studied by Forbis-Stokes et al. (2018). Also, the latter study had a large fraction of non-biodegradable COD. In Phase I, the COD contributed by wood chips was not necessary for the denitrification, but it was necessary for some denitrification in Phase II. When calculating denitrification rates based on the contribution of electron donors from wood chips alone, the denitrification rate in Phase II was either 0.030 kg_N_ m^−3^ d^−1^ when using a 4:1 COD:NO_3_–N ratio or 0.066 kg_N_ m^−3^ d^−1^ when using a 4:1 BOD:NO_3_–N ratio. Due to high relative standard deviation of BOD in this study, BOD values are difficult to rely upon. However, the lower percent removal values of BOD compared to COD (Table 1, Table 2) indicates that some of the COD entering the submerged filter was recalcitrant. The previous study by Forbis-Stokes et al. (2018) found a rate of 0.056 kg_N_ m^−3^ d^−1^ for denitrification enabled by bamboo woodchips alone, nearly equaling the value calculated here for the BOD relationship.

The removal rates and efficiencies of the P filter were less than have been previously found for similar filters, particularly in Phase II. Our filter achieved (on average) 47% removal efficiency with 126 g P m^−3^ d^−1^ removal rate at 0.77 d EBRT (Phase I) and 9% removal efficiency with 10 g P m^−3^ d^−1^ removal rate at 1.54 d EBRT (Phase II). Christianson et al. (2017) achieved 80% and 26% removal efficiencies with 60 and 8.8 g P m^−3^ d^−1^ at 0.75 d EBRT and 98% and 89% removal efficiencies with 25 and 13 P m^−3^ d^−1^ at 2.13 d EBRT 8.8–48 and 25–133 g P m^−3^ d^−1^ for steel slag and mine drainage, respectively, when placed after a wood chip filter. The highest removal rate was 133 g P m^−3^ d^−1^ at 0.30 d EBRT with 58% removal efficiency. The adsorption capacity (23.2 g P kg^−1^ iron), though, was on par with other media. A recent study (Pap et al., 2020) with calcite-chitosan achieved 21.56 g P kg^−1^ chitosan. Dobbie et al. (2009) achieved 26 and 22 g P kg^−1^ removal using granular ochre and ochre pellets, respectively (65% iron). While the phosphorus filter reduced P as desired, the effluent quality was not improved. Effluent leaving this filter was much darker in color than liquid from preceding filters, adding a reddish-black hue. Some oil residue was on the iron shavings before start and although the shavings were washed, maybe they were not washed sufficiently. This oil may have contributed to color along with oxidation products of the iron. Christianson et al. (2017) found that having the iron filter after denitrification produced better overall results; however, the color issue here may require that the iron filter be used earlier in the system’s processes or have a polishing filter succeeding the iron filter, such as a sand filter.

The filtration system was low-cost, small footprint, and did not require any exogenous materials for nutrient removal during the 16 week study period. The total infrastructure (support structure, drums, piping, and aeration pump) cost was $627 USD. Filtration media itself was inexpensive, but the manual processing we required to obtain known uniform particle sizes resulted in a cost of $800 USD. The system footprint was 2.2 m^2^, and total occupied space was 4.7 m^3^ while a soil absorption field for this stream would require about 14–25 m^2^, depending on soil type (Schultheis, n. d.). The system was built and installed by a small work crew (2–4 laborers at a time) within two weeks. Once installed, air was the only input required and was supplied by a low-wattage (2.5 W) and low-cost ($10 USD) aquarium pump. The daily energy demand was 14.4 Wh (51.8 kJ) or 0.20 kWh/m^3^ treated which could easily be supplied by a small solar panel. For a sanitation system comprising an anaerobic digester and the filters as described herein, the aeration pump would be the only energy demand which is much lower than that of traditional wastewater treatment plants, which were estimated at 0.49 kWh/m^3^ (Gurung et al., 2018). No exogenous electron donors were supplied to the denitrification filters as the bamboo chips provided enough reducing equivalents and did not show any indication of requiring replacement. The iron in the phosphorus filter demonstrated a similar adsorption capacity as previous studies but was undersized in this application. Increasing the filter size or replacing the medium would have had negligible impact on the footprint or cost as it was by far the smallest filter and the scrap iron materials were obtained at no cost. This system was able to treat a total of 6000 L of anaerobic digester effluent that was highly concentrated.

## 5 Conclusion

A full-scale three-stage filter for nitrogen and phosphorus removal from anaerobic digester effluent was implemented in Madagascar. The design was based on prior laboratory studies of similar but scaled-down systems. All filter materials were sourced locally. The filters achieved high rates of nitrogen transformation (nitrification and denitrification) along with removing solids, organics, and phosphorus. Though the influent feed was not as expected with much of nitrogen in the feed coming in as organic N and not as NH_3_–N, the filters still removed 38–49% of total incoming nitrogen. Even if target reductions of nitrogen and phosphorus were not fully attained, the approach did achieve high rates of removal and demonstrated a path towards reaching those targets (e.g., with slightly larger filters) while utilizing a simple and low-cost approach. Nitrification, denitrification, and phosphorus removal found in this full-scale study expand upon and support previous lab-scale findings and demonstrate feasibility of upscaling solutions from the lab to the field. Based on this study, simple effluent filters can support nutrient removal for small-scale and onsite fecal sludge treatment systems.

## CRediT authorship contribution statement

**Aaron A. Forbis-Stokes:** Conceptualization, Methodology, Investigation, Formal analysis, Writing - original draft, Writing - review & editing. **Graham H. Miller:** Conceptualization, Writing - original draft, Writing - review & editing. **Armel Segretain:** Methodology, Resources, Writing - review & editing. **Felahasina Rabarison:** Methodology, Resources, Writing - review & editing. **Tojoniaina Andriambololona:** Methodology, Resources, Writing - review & editing. **Marc A. Deshusses:** Conceptualization, Writing - original draft, Supervision, Project administration, Funding acquisition, Writing - review & editing.

## Declaration of competing interest

The authors declare that they have no known competing financial interests or personal relationships that could have appeared to influence the work reported in this paper.
